# Supplementary figures and images for: Paths of Evolution of Progressive Anaplastic Meningiomas: A Clinical and Molecular Pathology Study
Source: J Pers Med. 2023 Jan 25;13(2):206. doi: 10.3390/jpm13020206 (PMC9965923; doi:10.3390/jpm13020206)

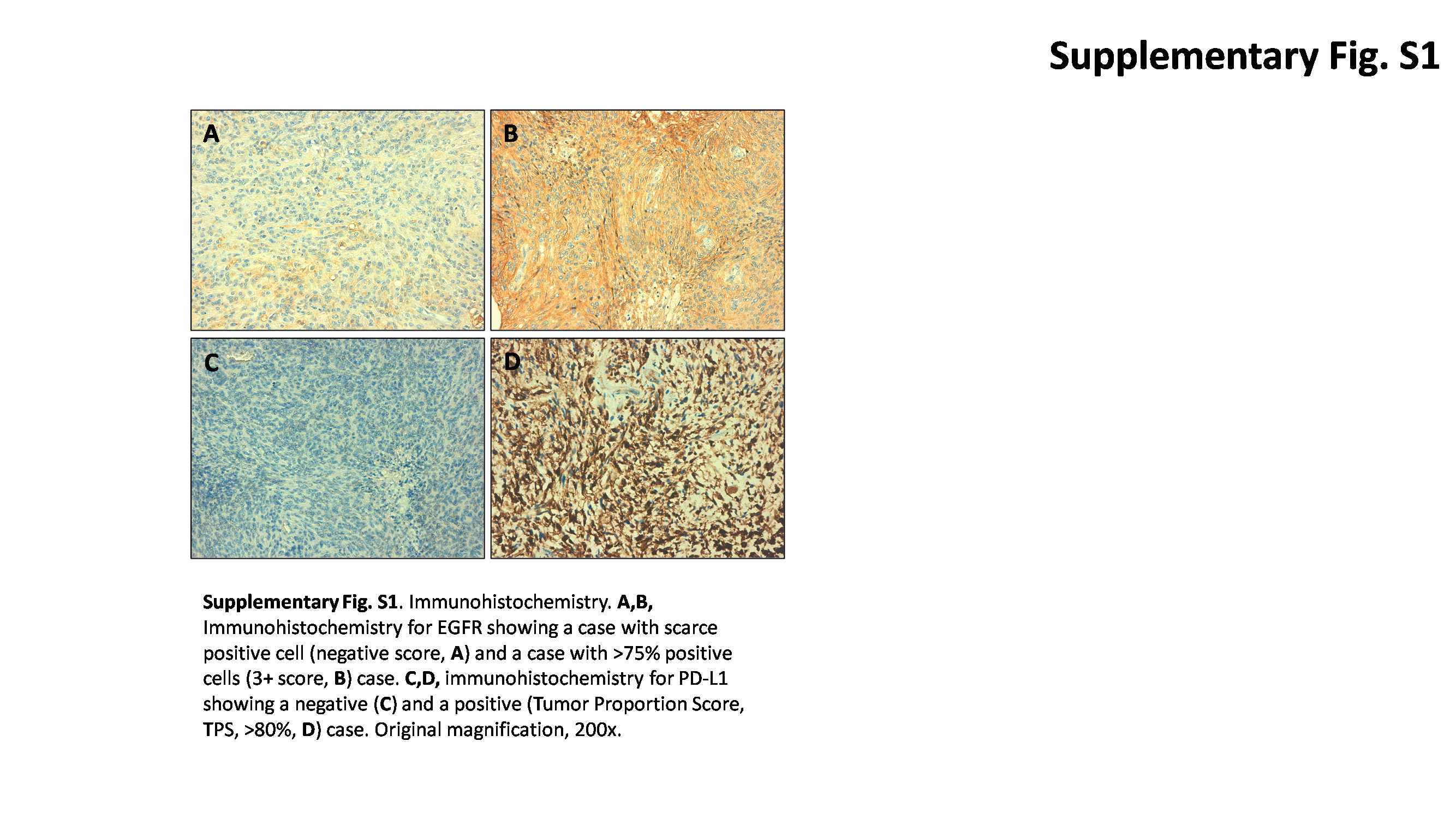

Supplement: Supplementary file 1 [file jpm-13-00206-s001.zip › Supplementary Figure S1.tif]

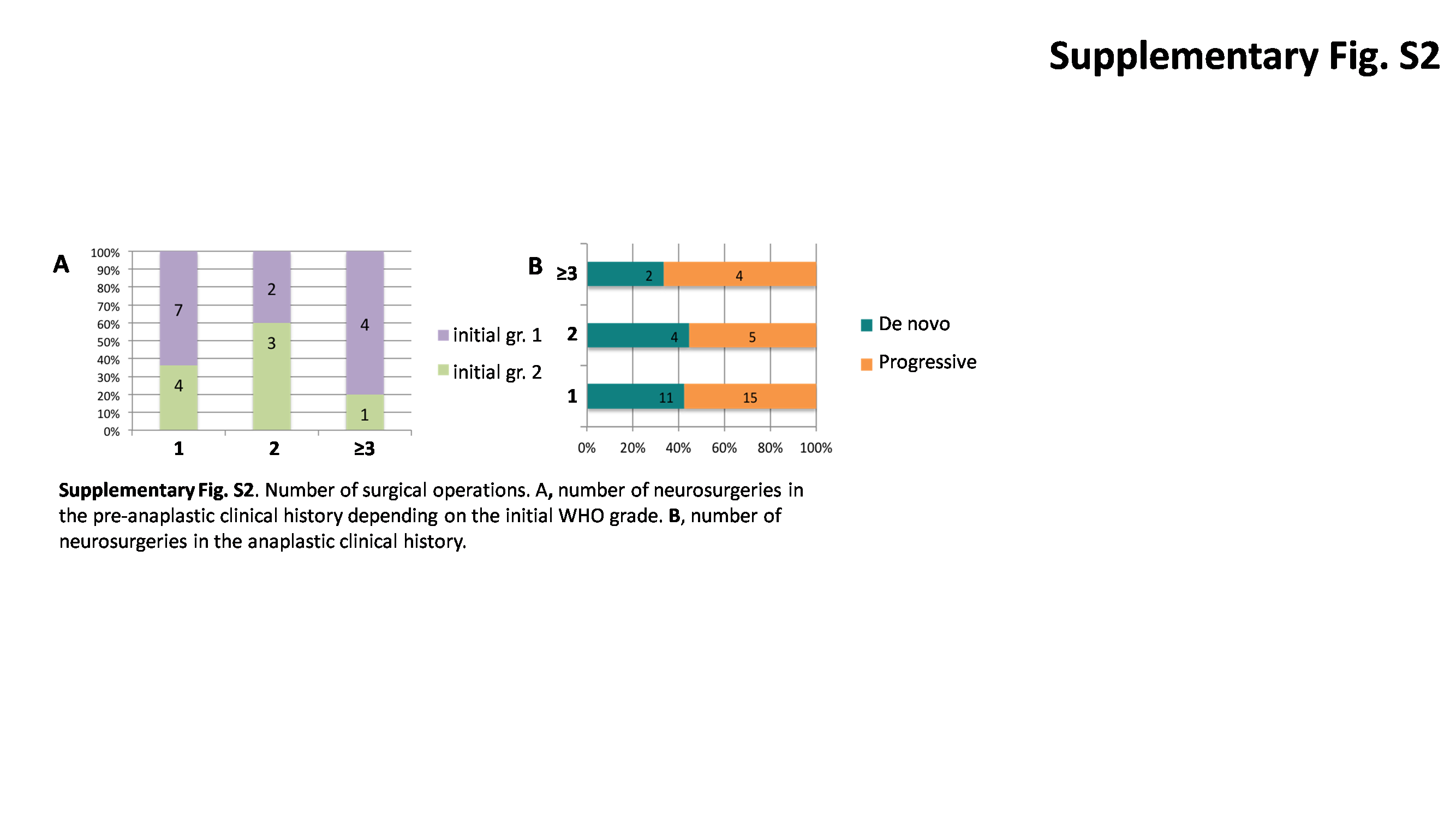

Supplement: Supplementary file 1 [file jpm-13-00206-s001.zip › Supplementary Figure S2.tif]
